# Associations between statin use and suicidality, depression, anxiety, and seizures: a Swedish total-population cohort study

**DOI:** 10.1016/S2215-0366(20)30311-4

**Published:** 2020-11

**Authors:** Yasmina Molero, Andrea Cipriani, Henrik Larsson, Paul Lichtenstein, Brian M D'Onofrio, Seena Fazel

**Affiliations:** a Department of Psychiatry, University of Oxford, Warneford Hospital, Oxford, UK; b Department of Medical Epidemiology and Biostatistics, Karolinska Institutet, Stockholm, Sweden; c Centre for Psychiatry Research, Department of Clinical Neuroscience, Karolinska Institutet, Stockholm, Sweden; d School of Medical Sciences, Örebro University, Örebro, Sweden; e Department of Psychological and Brain Sciences, Indiana University, Bloomington, IN, USA

## Abstract

**Background:**

Statins have shown both protective and adverse associations with neuropsychiatric outcomes. We aimed to examine the possible associations between statins and suicidality, depression, anxiety, and seizures.

**Methods:**

Using Swedish national registers, we linked data on dispensed statin prescriptions with data on unplanned (emergency) hospital visits or specialised outpatient care for four neuropsychiatric outcomes: suicidal behaviour (including deaths from suicide), depressive disorders, anxiety disorders, and seizures. We included all individuals in the registries who were dispensed statins and who were aged 15 years or older between Jan 1, 2006, and Dec 31, 2013. We applied a within-individual design using stratified Cox proportional hazards regression to compare the incidence of the defined outcomes during periods on statins and periods off statins within each individual, thus adjusting for time-invariant confounders. Non-specific effects of treatment were tested by investigating these outcomes in relation to thiazide diuretic use and antihistamine use in the same cohort.

**Findings:**

The statin-users cohort comprised 1 149 384 individuals, of whom 1 015 949 (88·4%) were aged 50 years or older, 625 616 (54·4%) were male, and 523 768 (45·6%) were female. The study period consisted of 2 053 310 non-treatment periods and 2 997 545 treatment periods, and 957 216 (83·3%) individuals had a medication status change (from on statins to off statins, or vice versa). Suicide outcomes were found in 6372 (0·6%) individuals, depressive disorders in 23 745 (2·1%), anxiety disorders in 30 100 (2·6%), and seizures in 28 844 (2·5%). There were no clear associations between periods of statin treatment and suicidal behaviour or deaths from suicide (hazard ratio 0·99 [95% CI 0·90–1·08]), anxiety disorders (0·99 [0·95–1·02]), or seizures (1·00 [0·97–1·04]). Statins were associated with reduced hazards of depressive disorders (0·91 [0·87–0·94]), which remained after adjustment for concurrent antidepressant use (0·91 [0·88–0·94]). Hazard ratios for depressive disorders were 0·61 (0·38–1·00; n=14 718) with thiazide diuretic use and 0·84 (0·67–1·06; n=23 715) with antihistamine use.

**Interpretation:**

Statin use is not associated with suicidality, anxiety disorders, or seizures. Whether the observed association between statin use and reduced diagnoses of clinical depression is confounded by non-specific benefits related to being prescribed medication needs further research.

**Funding:**

Wellcome Trust, Swedish Research Council, National Institute for Health Research (NIHR) Research Professorship, NIHR Oxford Health Biomedical Research Centre, American Foundation for Suicide Prevention, Karolinska Institutet.

## Introduction

Statins are among the most prescribed medications worldwide.^1^ Their antithrombotic, anti-inflammatory, and antioxidative effects have been widely examined,^2^ and they are recommended for primary and secondary prevention of cardiovascular events.^3^ However, concerns have been raised about the potential neuropsychiatric adverse effects of statins, including increased anxiety,^4^ depression,^5^ and suicidality.^4^ These concerns have been mainly based on case series and a few observational studies. By contrast, case-control studies and randomised controlled trials suggest no such associations.^6^, ^7^ Evidence also suggests a beneficial effect of statins on depression when used as an add-on treatment with SSRIs.^8^, ^9^, ^10^, ^11^ Furthermore, statins alone (ie, without adjunctive SSRI treatment) might have a protective effect against depression, psychiatric hospitalisation, and suicidal behaviour,^7^, ^12^, ^13^, ^14^ although results are mixed.^15^, ^16^, ^17^ Statins might also decrease the risk of seizures,^18^, ^19^, ^20^ although some evidence suggests no such effect.^21^, ^22^

The differential effects of statins on depression and seizures have been attributed to structural differences between statin classes;^18^, ^22^ however, few comparisons between classes are available. Contrasting findings on the association between statins and neuropsychiatric outcomes could also be attributed to differences in study design, the extent of adjustment for confounding factors, choice of outcome measure, or inadequate sample sizes. Clarification of these associations could have important implications: for mental health, if a protective effect is replicated in real-world settings, it would underscore the need for intervention trials; and also for public health, as unwarranted concerns about safety have been associated with decreased statin use.^23^

**Research in context**

**Evidence before this study**

Hydroxymethylglutaryl coenzyme A (HMG-CoA) reductase inhibitors, or statins, are commonly prescribed for primary and secondary prevention of cardiovascular events. They have been linked to both beneficial and adverse neuropsychiatric outcomes, including an increased risk of anxiety, depression, and suicidality, as well as seizures. We searched PubMed from Jan 1, 2000, to March 15, 2020, using the search terms “statins”, “HMG-CoA reductase inhibitors”, “suicid*”, depressi*”, “anxiety”, “epilep*”, “seizure*”, “psychiatr*”, “neuropsychiatr*”, “aggression*”, and “violen*”, with no language restrictions. Results were inconsistent, with individual studies variously reporting increased or decreased risks of neuropsychiatric outcomes and some reporting no associations. Furthermore, some evidence from observational studies and randomised controlled trials showed a beneficial effect of statins on depression when used as add-on treatment to antidepressants. Systematic reviews of randomised control trials showed short-term effects of treatment, but there was insufficient information to draw hypotheses regarding the timing of outcomes.

**Added value of this study**

This study addressed limitations in previous research by examining a population-based cohort of more than a million individuals treated with statins, using nationwide registry data to examine associations between statin use and four neuropsychiatric outcomes: suicidal behaviour and deaths from suicide, depressive disorders, anxiety disorders, and seizures. We applied a within-individual design that controls for time-invariant confounders (such as genetics and psychiatric history), and more fully adjusts for stable factors associated with confounding by indication in observational data (ie, that the reason for prescribing the medication is also associated with the outcome) than between-individual designs. Results showed no clear associations between statin treatment and suicidal behaviour and deaths from suicide, anxiety disorders, or seizures. Statins were associated with reductions in depressive disorders, which remained when controlling for antidepressant use. However, rates of depressive disorders were also reduced (albeit non-significantly) when two control medications (thiazide diuretics and antihistamines) were used as independent exposure medications in the statin-users cohort.

**Implications of all the available evidence**

Evidence from randomised controlled trials and high-quality observational studies suggest that statin treatment is a safe therapeutic option with regard to suicidal outcomes, anxiety disorders, seizures, and depression. The observed reduction in depression rates reported in some studies should prompt further investigation into the possible contributions of non-specific treatment factors.

To address uncertainties in previous research, we aimed to examine a large population-based cohort of statin users, and assess four neuropsychiatric outcomes—suicidal behaviour and deaths from suicide, depressive disorders, anxiety disorders, and seizures—that have been linked to both beneficial and adverse outcomes. We used a within-individual design that controls for time-invariant confounders such as genetics and psychiatric history, and more fully adjusts for stable factors associated with confounding by indication in observational data (ie, that the reason for prescribing the medication is also associated with the outcome) than between-individual designs.

## Methods

### Study design and participants

We did a population-based longitudinal cohort study using Swedish nationwide registers linked through personal identification numbers, and applied a within-individual design^24^ accounting for time-invariant confounders.

We identified all individuals in the Swedish population who were dispensed statins (ie, had filled-in and collected prescriptions) and were aged 15 years or older at any time during the study period. The study period was delimited on the basis of data availability in the registers. Data on medication exposure in the Prescribed Drug Register was available from July 1, 2005, and our study period started on Jan 1, 2006, to ensure that we captured the actual start of a treatment period. Thus, we excluded all treatments that started before Jan 1, 2006, to have a 6-month time window that was treatment-free. Our study period ended on Dec 31, 2013, as this was the last available date for the register linkage.

The project was approved by the regional ethics review board in Stockholm, Sweden (approval numbers 2013/5:8), which waived the need for informed consent.

### Medications

Statins were defined as hydroxymethylglutaryl coenzyme A reductase inhibitors (Anatomical Therapeutic Chemical classification system C10AA). In Sweden, all citizens are insured with common non-claim health-care insurance with subsidised medications. Data on dispensed medications were extracted from the Swedish Prescribed Drug Register, which includes information on all prescriptions dispensed from all pharmacies in Sweden since July, 2005, and has less than 0·3% missing information. Full details are provided in the appendix (p 2). As the Swedish pharmaceutical benefits scheme allows for a maximum of 3 months' supply for each prescription, treatment periods were defined as at least two consecutive dispensations within 6 months to ensure treatment continuity. Dispensations more than 6 months apart from the last dispensation were considered to be the start of a new treatment period. Each treatment period started at the date of the first dispensation, and ended at the date the last prescription was dispensed within that treatment period. Individuals with other treatment patterns (ie, individuals who filled a single prescription or with irregularly filled prescriptions, where each prescription filled was more than 6 months after the previous one) were not included in the analyses because of uncertainty over medication adherence. However, we also did sensitivity analyses including only these individuals. Statins were first analysed as a whole class, and then by individual statin (appendix p 2).

### Neuropsychiatric outcomes

Neuropsychiatric outcomes included unplanned (ie, emergency) hospital and specialised outpatient care visits due to the following: self-injurious behaviour or suicide attempt, or death from suicide (ICD-10 codes X60–X84); depressive disorders (F32–F34, F38–F39); anxiety disorders (F40–F45, F48); or seizures (G40–G41, R56). These data were extracted from the Swedish Patient Register and Cause of Death Register. Full details are provided in the appendix (p 2).

### Statistical analyses

All participants were followed up from the start of the study period, or the date of immigration to Sweden, and were censored at end of study period, or at death or permanent emigration (ie, migration from Sweden without returning before the end of the study period). All time was split into periods of treatment and non-treatment. We removed periods in which medication exposure or outcomes might not have been captured in the registers, such as periods of emigration, prison (collected from the Prison and Probation Services Register, and including 2613 individuals [0·2% of the cohort]), and hospitalisation, to account for time at risk.

A within-individual design using stratified Cox proportional hazards regression was applied to examine associations between statins and outcomes. This form of self-controlled case series compares periods on medication to periods off medication within each individual. The reason for using a within-individual design rather than a statistical matching technique (eg, propensity score matching) was that it allowed us to control for time-invariant confounders that are not readily observed in registry data (such as genetic and historical factors, and factors at the start of treatment),^24^ as each individual serves as their own control. Compared with between-individual designs, the within-individual design also more fully adjusts for confounding by indication that is stable during the study period. However, this design does not adjust for time-varying factors (ie, factors that change during follow-up, such as age, health status, changes in indication, or use of other medications). In the within-individual design, all individuals in the cohort are included in the analyses. However, only individuals who change medication status (ie, from on treatment to off treatment or vice versa) and who have an outcome contribute directly to the estimate of medication exposure on the outcome. All other individuals contribute indirectly, through their contribution to the estimate of the association with time-varying covariates that are adjusted for in the analyses (appendix p 3). We adjusted for age as a continuous time-varying covariate in all our analyses.

In sensitivity analyses, to adjust for the effect of concurrent antidepressant use in the association between statins and depressive disorders, we introduced concurrent treatment with antidepressants as a time-varying covariate. We repeated this with stratification by type of antidepressant (ie, SSRIs and all other antidepressants). Definitions of antidepressants are provided in the appendix (p 2).

To test the robustness of results, we examined associations between statin treatment and depressive disorders by using primary care data (rather than hospital and specialised outpatient care data) from Stockholm county (appendix p 2). In these analyses, we included individuals from the main cohort who had been prescribed statins within Stockholm county, and examined depressive disorders diagnosed by Stockholm county primary care providers. Furthermore, we examined associations between statin use and severe depression by including only inpatient treatment for depressive disorders. We also accounted for a potential overestimation of diagnoses in outpatient secondary care (ie, that diagnoses from recent visits might be recorded during new emergency visits) by excluding diagnoses within 90 days of a previous diagnosis. To account for a potential underestimation of depressive disorders in our main analyses (in which we included unplanned or emergency visits only), we also did analyses in which all visits (both emergency and planned) due to depressive disorders were included.

We also examined associations between statin use and depressive disorders on the basis of dose exposure (as measured by the defined daily dose; appendix pp 2–3). In addition, we examined whether treatment duration was differentially associated with depressive disorders by comparing long-term (longer than the median treatment duration) and short-term (median or less) treatment periods to periods of no treatment.

In our main analyses, we defined the end of a treatment period as the date of the last prescription within that period. To account for the possibility that participants take their medications for up to 3 months after their last filled prescription (as the Swedish pharmaceutical benefits scheme would allow), we repeated the main models but with the definition of treatment periods extended to 3 months after the last filled prescription.

To account for prevalent user bias (ie, that a proportion of participants were exposed to statins before the start of the study period and were not liable to adverse effects in the early phase of treatment, which could bias the association between statins and depression), we included a wash-out period of 24 months, examining only those who had been treatment-free between July 1, 2005, and July 1, 2007.

To address the possibility of non-specific treatment effects in individuals with a history of depressive disorders (eg, increased supervision during statin treatment because of concern over adverse events), we did analyses excluding all individuals with a history of depressive disorders before the start of the study period.

Our main models excluded individuals with single or irregularly filled prescriptions, because of uncertainty over treatment adherence. To address a potential for selection bias, we did sensitivity analyses including only these individuals. In these analyses, we defined a treatment period as the 90 days following a filled prescription.

To control for the potential effect of β blockers and angiotensin-converting enzyme (ACE) inhibitors on depression, we did analyses excluding individuals who had been prescribed β blockers or ACE inhibitors (appendix p 2), respectively, during the study period. We also used thiazide diuretics and antihistamines (appendix p 3) as independent exposures in the statins cohort to examine non-specific treatment effects.^25^

In further sensitivity analyses, stratifications were made by specific statin class (atorvastatin, pravastatin, rosuvastatin, and simvastatin). Finally, we examined arrests for violent and non-violent crime (appendix p 2) to test for associations with other outcomes related to impulsivity.

For all analyses, 95% CIs are presented. We used SAS version 9.4 and STATA version 14.1. The Strengthening the Reporting of Observational studies in Epidemiology (STROBE) reporting guidelines were followed (appendix pp 8–9).

### Role of the funding source

The funder of the study had no role in study design, data collection, data analysis, data interpretation, or writing of the report. YM and SF had full access to all the data in the study and had final responsibility for the decision to submit for publication.

## Results

From the total population of Sweden aged 15 years or older (n=8 945 599), we identified 1 149 384 (12·9%) individuals who had been treated with statins between 2006 and 2013 (table 1). Among this statin-users cohort, 625 616 (54·4%) were males and 1 015 949 (88·4%) were 50 years or older at the start of the study (table 1). The most prescribed statin class was simvastatin, prescribed to 1 006 490 (87·6%) of the statin-users cohort. During the study period, 6372 (0·6%) statin users had suicidal outcomes (suicide attempts and deaths from suicide), 23 745 (2·1%) were diagnosed with a depressive disorder, 30 100 (2·6%) were diagnosed with an anxiety disorder, and 28 844 (2·5%) were treated for seizures. 718 575 (62·5%) started the study period as non-treated. The median number of treatment periods was 3 (IQR 2–5), and the median duration of a treatment period was 293 days (104–728). Descriptive data are provided in table 1, and stratified by statin class in the appendix (p 5).

**Table 1:** Descriptive data for individuals dispensed statins during the study period (2006–13)

	**Participants (n=1 149 384)**
**Sex**	
Male	625 616 (54·4%)
Female	523 768 (45·6%)
**Age at start of study period, years**	
<40	31 879 (2·8%)
40–49	101 556 (8·8%)
50–59	254 997 (22·2%)
60–69	374 424 (32·6%)
70–79	276 764 (24·1%)
≥80	109 764 (9·5%)
Statin classes dispensed*	
Simvastatin	1 006 490 (87·6%)
Atorvastatin	244 482 (21·3%)
Rosuvastatin	54 080 (4·7%)
Pravastatin	41 012 (3·6%)
Fluvastatin	6817 (0·6%)
**Individuals with outcome event during study period**	
Suicidal behaviour and deaths from suicide	6372 (0·6%)
Depressive disorders (emergency visits only)	23 745 (2·1%)
Depressive disorders (both planned and unplanned visits)	53 395 (4·6%)
Anxiety disorders	30 100 (2·6%)
Seizures	28 844 (2·5%)
Arrests for violent crime	6720 (0·6%)
Arrests for non-violent crime	45 054 (3·9%)
**Number of events during study period (during non-treatment periods; during treatment periods)**	
Suicidal behaviour and deaths from suicide	7432 (3728; 3704)
Depressive disorders	41 718 (18 400; 23 318)
Anxiety disorders	44 745 (19 698; 25 047)
Seizures	45 587 (14 102; 31 485)
**Incidence during study period, per 1000 person-years**	
Suicidal behaviour and deaths from suicide	1·0
Depressive disorders	5·6
Anxiety disorders	6·0
Seizures	6·1
**Medication status during study period**	
Number of non-treatment periods	2 053 310
Number of treatment periods	2 997 545
Individuals not medicated at start	718 575 (62·5%)
Individuals medicated at start	430 807 (37·5%)
Individuals with at least one medication status change	957 216 (83·3%)
**Individuals with outcome event among those with medication status change (N=957 216)**†	
Suicidal behaviour or death from suicide	3979 (0·4%)
Depressive disorder	20 363 (2·1%)
Anxiety disorder	20 412 (2·1%)
Seizure	15 553 (1·6%)
**Treatment patterns**	
Median number of treatment periods (IQR)	3 (2–5)
Median number of days in a treatment period (IQR)	293 (104–728)
**Other medications during the study period**	
Antidepressants	333 850 (29·1%)
β blockers	648 317 (56·4%)
Angiotensin-converting enzyme inhibitors	484 662 (42·2%)

Data are n (% of cohort), unless otherwise specified.

* Statin classes were not mutually exclusive; 203 497 individuals (17·7% of the cohort) were treated with at least two different statin classes during the study period.

† Refers to individuals who had at least one event of the outcome in question, out of those who changed medication status at least once during the study period (from on treatment to off treatment or vice versa).

We did within-individual analyses comparing all treatment periods to all non-treatment periods (figure). Total numbers of treatment and non-treatment periods are shown in table 1. Periods of statin treatment were associated with lower rates of depressive disorders (hazard ratio [HR] 0·91 [95% CI 0·87–0·94]). No statistically significant associations were found for self-injurious or suicidal behaviour (0·99 [0·90–1·08]), anxiety disorders (0·99 [0·95–1·02]), or seizures (1·00 [0·97–1·04]).

**Figure:**
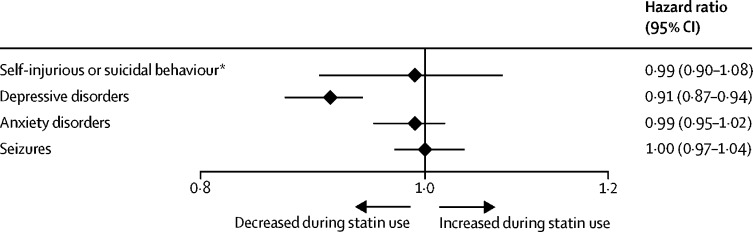
Associations of neuropsychiatric outcomes with periods of statin use from within-individual analyses *Includes suicide attempts and deaths from suicide.

In sensitivity analyses adjusting for the possible confounding effect of antidepressant medication use, associations between statin use and depressive disorders remained similar to those found in the overall analyses, whether considering all antidepressant medications, or SSRI and non-SSRI antidepressants separately (table 2).

**Table 2:** Sensitivity analyses of within-individual associations between periods of statin use and depressive disorders

	**Number in sample**	**Number of events**	**Hazard ratio (95% CI)**
**Adjusting for concurrent antidepressant treatment**			
Any antidepressant treatment	1 149 384	41 718	0·91 (0·88–0·94)
SSRI treatment	1 149 384	41 718	0·91 (0·88–0·95)
Non-SSRI antidepressant treatment	1 149 384	41 718	0·91 (0·88–0·95)
**Alternative samples**			
Stockholm county primary care data	203 711	40 778	0·94 (0·91–0·97)
Excluding individuals with depressive disorders before the start of the study period	1 133 660	31 658	0·92 (0·88–0·96)
Excluding individuals with β blockers	501 065	16 870	0·92 (0·87–0·98)
Excluding individuals with angiotensin-converting enzyme inhibitors	664 720	23 577	0·91 (0·86–0·95)
Including only individuals who were treatment-free for 24 months before first statin treatment	510 904	21 191	0·92 (0·87–0·97)
Including only individuals with single or irregular dispensations	121 638	3576	0·96 (0·76–1·22)
**Alternative outcomes**			
Inpatient treatment only	1 149 384	26 714	0·87 (0·83–0·91)
Excluding diagnoses within 90 days	1 149 384	32 100	0·91 (0·87–0·96)
Including diagnoses from both planned and unplanned visits	1 149 384	179 678	0·96 (0·94–0·97)
**Alternative exposures**			
Statin dose (number of defined daily doses)	1 149 384	41 718	..
Low (<1)	..	..	0·94 (0·90–0·97)
Moderate (1–2)	..	..	0·89 (0·84–0·93)
High (>2)	..	..	1·00 (0·92–1·09)
Statin treatment duration	1 149 384	41 718	..
Long (>293 days)	..	..	0·91 (0·87–0·95)
Short (≤293 days)	..	..	0·90 (0·83–0·97)
End of treatment period 3 months after last filled prescription	1 149 384	41 718	0·94 (0·91–0·97)
Thiazide diuretics as exposure	14 718	2773	0·61 (0·38–1·00)
Antihistamines as exposure	23 715	25 975	0·84 (0·67–1·06)

We further tested the association between statin use and depressive disorders using primary care data, which included 203 711 (17·7%) individuals from the statin-users cohort, of which 8315 (4·1%) had a depressive disorder outcome. Depressive disorder outcomes were decreased during periods of statin use compared with periods of no statin use, similar to the analyses based on national hospital and specialised outpatient care data (table 2). When we examined hospital inpatient treatment only (as a proxy for depression severity), excluded diagnoses within 90 days of a previous diagnosis, or included all visits (both emergency and planned visits) due to depressive disorders, results remained similar to those of the main analysis (table 2).

Further sensitivity analyses showed that periods of statin use at low doses (16 049 126 [60·2%] treatment intervals) or moderate doses (8 338 040 [31·3%] treatment intervals) were associated with reductions in depressive disorders compared with periods of no statin treatment; however, periods of high-dose statin use (2 251 179 [8·5%] treatment intervals) showed no statistically significant association (1·00 [0·92–1·09]; table 2). Hazards were similar whether long-term (>293 days) or short-term (>293 days) treatment periods were considered as the exposure, and when the end of a treatment period was extended to 3 months after the last filled prescription (table 2).

When we included a wash-out period by examining only those who had been treatment-free for at least 24 months before starting their first statin treatment (n=510 904 individuals), and when we excluded all individuals with a history of depressive disorders before the start of the study period (n=1 133 660), results were similar to the overall findings, showing reduced depressive disorder outcomes during periods of statin use compared with periods of no statin use (table 2).

To address a potential for selection bias in our statin-users cohort, we examined associations in individuals who were not included in the cohort due to uncertainty over treatment continuity (ie, those with single or irregularly filled prescriptions; n=121 638). No association between statin use and depressive disorders was found in this group (HR 0·96 [95% CI 0·76–1·22]). Analyses excluding individuals who were treated with β blockers or ACE inhibitors during the study period showed results similar to those of the main analysis (table 2).

We used two negative controls in the statin-users cohort to account for non-specific treatment effects. Hazard ratios for thiazide diuretic use (0·61 [0·38–1·00], n=14 718) and antihistamine use (0·84 [0·67–1·06], n=23 715) indicated reduced rates of depressive disorder outcomes, although these associations did not reach statistical significance.

When investigating each statin class separately (appendix pp 4–5), depressive disorder outcomes were reduced during periods of pravastatin (HR 0·72 [95% CI 0·53–0·99]) and simvastatin treatment (0·87 [0·83–0·90]); and anxiety disorder outcomes were reduced during atorvastatin (0·89 [0·81–0·97]), pravastatin (0·73 [0·56–0·95]), and rosuvastatin treatment (0·84 [0·70–0·99]).

Finally, because we considered that there was likely to be under-reporting of suicidal outcomes, we tested the robustness of associations between statin use and suicidal behaviour by examining two other outcomes related to impulsivity. Results showed that arrests for violent crimes (HR 0·81 [95% CI 0·73–0·89]) and for non-violent arrest (0·90 [0·88–0·93]) were reduced during periods of statin treatment (appendix p 6).

## Discussion

In this national cohort study, we identified 1 149 384 individuals aged 15 years or older who had been treated with statins between 2006 and 2013 in Sweden, which amounts to around 13% of the population in the same age group. Using a within-individual design, we found no clear associations between statin use and suicidal outcomes, anxiety disorders, or seizures. Periods on statin treatment were associated with a lower risk of diagnosed depressive disorders, and this reduction remained after adjustment for concurrent antidepressant use. When individuals were prescribed thiazide diuretics or antihistamines (which acted as negative controls) in the statin-users cohort, hazard ratios indicated reduced risk of depressive disorder outcomes, although statistical significance was not reached, suggesting that the depression findings might be partially confounded. Data from randomised controlled trials and observational studies suggest that statins are associated with a 25–35% lower risk of depressive symptoms and diagnosis when used as an add-on to SSRI treatment.^9^, ^10^, ^26^ The rate reductions were smaller in our sample at 9%, which could be explained by differences in outcome measures (eg, randomised controlled trials using symptom scales) or in design (eg, not accounting for confounding by indication in observational studies, and the current study not solely investigating people with baseline depression). Our results on reduced risk when adjusting for concurrent antidepressant use are consistent with a meta-analysis of randomised controlled trials,^13^ and a meta-analysis of observational studies of statin treatment alone (without adjunctive SSRIs),^12^ although the reductions seen in those studies were greater, at around 30%. Again, differences in design, baseline depressive symptoms, and outcome measures could explain the smaller associations seen in our study. Additionally, we found reduced depressive outcomes when excluding individuals who had also been treated with β blockers and ACE inhibitors, other cardiovascular medications linked to depression.

Large observational studies have reported no associations between statins and depression.^27^, ^28^, ^29^ The contrast between those findings and our results could be explained by differing measures of depression (eg, two studies used antidepressants as a proxy for depression, and another study combined bipolar disorder, depression, and post-traumatic stress disorder into one measure). Importantly, all those studies used between-individual models, comparing individuals prescribed statins to those not prescribed statins, which can only adjust for factors that are measured. Consequently, compared with the within-individual design, such models are less able to adjust for stable factors associated with confounding by indication that are not readily observed in the data.

Furthermore, some statin classes (simvastatin and pravastatin) were associated with reduced risk of depressive disorders, whereas others (atorvastatin and rosuvastatin) were not, although these latter findings were limited by the small sample size. 88% of participants in our cohort were prescribed simvastatin, and the observed associations found when analysing all statins as a single class might have been largely driven by the reductions observed for simvastatin. Some evidence points to simvastatin having a stronger effect on depressive symptoms,^9^ although there have been some contrasting findings.^30^ Statins differ in their ability to cross the blood–brain barrier, and it has been suggested that statins with higher blood–brain barrier permeability (such as simvastatin) might have greater antidepressant effects, because they can decrease inflammation locally in the brain.^20^, ^26^ However, only a small number of head-to-head comparisons have been done, and more research is needed.

The antidepressant effects of statins have been suggested to be mediated by their pleiotropic effects, including inhibition of inflammatory responses, reduction of oxidative stress, and modulation of some cytokines.^31^ Another suggestion is that decreased cardiovascular risk, increased health consciousness, and treatment compliance could improve quality of life, thereby decreasing the risk of depression.^12^, ^13^ However, the evidence base is still small, and it is not known which mechanism could be responsible for the potential protective effects of statins. When examining associations between risk of depressive disorder and statin dose exposure, we found reduced risk during periods of exposure to low or moderate statin doses (more than 90% of statin treatment intervals), but not for periods of high-dose exposure, suggesting that there was no dose–response association between statins and depression. When we used thiazide diuretics or antihistamines as negative controls in the statin-users cohort (independent of concurrent statin use), we found reduced risks of depressive disorders, but this association was not significant. This lack of significance might be explained by the smaller sample sizes compared with that of the main analysis, but was consistent across these two medications. Nevertheless, one interpretation of our findings could be that reductions in depression are explained by non-specific treatment factors rather than a direct neuroprotective mechanism. Consistent with this interpretation, we found no associations between statin use and depressive disorders in individuals with single or irregular dispensations (ie, where treatment continuity was uncertain).

Depression has been suggested to reduce adherence to medication in individuals with cardiovascular events.^32^ Thus, individuals in our study might have been more likely to use statins when they were not depressed, which could mean that the causality is actually reversed. Similarly, treating physicians might treat physical health problems differently in individuals with and without depressive disorders, which could result in fewer statin prescriptions when individuals present with depressive symptoms. Statin use was associated with stronger reductions when the outcome considered was inpatient treatment for depression (assumed to represent more severe cases of depression) than when the analysis was based on patients treated in a primary care setting (Stockholm Country primary care data), or, similarly, when we included only emergency visits (as opposed to all visits, both planned and emergency) as the outcome. These findings could either support a neuroprotective mechanism for statins, or show that adherence is stronger for more severe depression. In older people, the mechanisms for depression might be driven by different biological factors, such as vascular diseases,^33^ and the potential contribution of inflammatory processes could also differ by age group. As such, it might be that statins have more benefit to younger individuals or to individuals who have specific anti-inflammatory pathways, which would not be apparent using large population-based data. Tailoring treatments for depression will require investigation of such subgroups, and future research should consider these differential effects of novel pharmacotherapies.

We found no associations between statins and seizures, consistent with the results from two large propensity score-matched cohorts.^21^ Statins were also not associated with anxiety disorders, in keeping with five randomised controlled trials^17^ that reported no differences in anxiety levels for individuals treated with simvastatin. Furthermore, our results do not support an association between statins and suicidality risk, consistent with a nationwide Danish observational study that showed no increased risk of suicide among patients treated with SSRIs and adjunctive statins compared with patients treated with SSRIs alone.^9^ The lack of association with suicidality in our study was supported by two other outcomes related to impulsivity—arrests for violent and for non-violent crime—for which we found a decreased risk during periods of statin use. Without further research into mechanisms, the explanation for this reduction is unclear. As with depression, the non-specific effects of treatment might be relevant. However, another cohort study using Swedish health registers^34^ reported that statin use was associated with reduced suicidal behaviour. Our different findings could be due to differences in the samples: while the previous Swedish study included only individuals with severe mental disorders (ie, bipolar disorder, schizophrenia, and non-affective psychosis), we included all individuals treated with statins.

This study has several strengths, including an 8-year study period; a large, population-based cohort of more than a million individuals treated with statins; nationwide coverage; inclusion of clinical outcomes from validated, high-quality registers; and complete information on statin dispensations, as each filled-in prescription was registered. Furthermore, we used a within-individual design, thus controlling for time-invariant covariates.

However, several limitations should be taken into account when interpreting the results. Importantly, this was an observational study that principally examined associations, and caution should be exercised when drawing causal inferences. In addition, although our model more fully adjusts for stable factors associated with confounding by indication in observational data than between-individual models, it does not account for confounders that could change during treatment (eg, time-varying factors such as psychiatric or somatic comorbidity, changes in indication, exercise, or lifestyle).

We did not have information on medication adherence; however, to address this limitation, we only included individuals with at least two dispensed prescriptions within 6 months. Our results could also be affected by a potential bias towards the null due to misclassification of medication exposure. Furthermore, our measure of treatment discontinuation was based on a conservative assumption: the date of the last collected prescription. This assumption could result in slightly lower sensitivity, thus underestimating associations. However, we did sensitivity analyses in which we extended the end of the treatment period to 3 months after the last filled prescription, and these results showed similar associations. Another limitation is that the use of official registers could involve selection effects and could underestimate rates of underlying disorders and outcomes. On the other hand, the registers capture information on health-care contacts, reflecting real-world outcomes that consume resources.^35^

The Prescribed Drug Register started in July, 2005, and previous prescriptions were not recorded. Although we excluded treatment periods before this date and the start of the study period (Jan 1, 2006), participants could have been treated with statins before that time. In a sensitivity analysis, we included a wash-out period of 24 months (ie, we examined only those who had been treatment-free for at least 24 months before starting statin treatment), and results were similar to those of the main analysis.

Finally, differences in prescription practices and outcome prevalence between countries might affect the generalisability of findings. The prevalence of depressive disorders in Sweden is similar to that in other countries (around 5%).^36^ However, the prevalence of depressive disorders was lower in our study (2·1%), probably because of methodological reasons: we included only unplanned (emergency) visits to hospitals and specialised outpatient care. When including all visits (both planned and emergency), the prevalence of depressive disorders (4·7%) was similar to the prevalence in the general population. Our use of a more conservative measure of depressive disorders (unplanned visits only) could potentially underestimate associations. Statin use was, however, associated with reductions in depressive disorders when using both outcome measures. Finally, although prescription sales of statins increased in Sweden during the study period, the increase was smaller compared with that in 12 other western European countries, and statin use in Sweden was below average in 2012 (mean defined daily doses per 1000 inhabitants per day was 95 in Europe, and 76 in Sweden), which needs to be considered for generalisability.^37^

In summary, we found no associations between statin treatment and suicidal outcomes, anxiety disorders, or seizures, suggesting that statin treatment is a safe therapeutic option with regard to some neuropsychiatric outcomes. We observed a reduction in the risk of depressive disorders during periods of statin treatment, but a similar (albeit non-significant) reduction was seen during treatments with two other non-psychotropic medications, and this association requires further investigation to clarify the possible contribution of non-specific treatment factors.
