# Supplementary material for: Associations between statin use and suicidality, depression, anxiety, and seizures: a Swedish total-population cohort study
Source: Lancet Psychiatry. 2020 Nov;7(11):982–90. doi: 10.1016/S2215-0366(20)30311-4 (PMC7606915; doi:10.1016/S2215-0366(20)30311-4)
Supplement: Supplementary appendix [file mmc1.pdf]

THE LANCET
Psychiatry

Supplementary appendix

This appendix formed part of the original submission and has been peer reviewed. We post it as supplied by the authors.

Supplement to: Molero Y, Cipriani A, Larsson H, Lichtenstein P, D'Onofrio BM, Fazel S. Associations between statin use and suicidality, depression, anxiety, and seizures: a Swedish total-population cohort study. *Lancet Psychiatry* 2020; **7:** 982–90.

SUPPLEMENTARY APPENDIX TO THE MANUSCRIPT

Associations between statin use and suicidality, depression, anxiety, and seizures: a Swedish total-population cohort study

Yasmina Molero, Andrea Cipriani, Henrik Larsson, Paul Lichtenstein, Brian M. D'Onofrio, Seena Fazel

Contents

Supplementary methods 2
Registers 2
Statins 2
Neuropsychiatric outcomes 2
Crimes 2
Primary care diagnoses 2
Other medications 2
Defined Daily Doses 2
Negative controls 3
Stratified Cox proportional hazards regression 3
Appendix Figure 1 4
Appendix Table 1 5
Appendix Table 2 6
References 7
STROBE Statement—Checklist 8

SUPPLEMENTARY METHODS

Registers

Registers included the Total Population Register, the Swedish Prescribed Drug Register, the Swedish Patient Register, the Cause of Death Register, the Stockholm Primary Care Register, the Register of Persons Suspected of Offences, and the Prison and Probation Services Register.[1-6]

Statins

Information on statins was collected from the Swedish Prescribed Drug Register,[5] and included HMG CoA reductase inhibitors (Anatomical Therapeutic Classification [ATC] System: C10AA), and included simvastatin (ATC: C10AA01), pravastatin (ATC: C10AA03), atorvastatin (ATC: C10AA05), rosuvastatin (ATC: C10AA07), and fluvastatin (ATC: C10AA04). Statins were first analysed as a whole class, and then separately. However, fluvastatin (ATC: C10AA04) was not analysed as a separate class, as there were too few individuals in this cohort to allow for separate analyses.

Neuropsychiatric outcomes

Information on all outcomes in the main analyses was collected from the Swedish Patient Register.[3] This includes all admissions to all hospitals in Sweden, as well as all outpatient contacts with specialized secondary care. The primary diagnosis is listed in 99% of all hospital discharges, and the positive predictive value of medical diagnoses in this register ranges between 85-95%.[3] In a previous register-based study, depression diagnoses were validated by comparing concordance rates with another clinical register that was based on multidisciplinary inpatient assessments, to make diagnoses as a gold standard. Results showed fair to moderate agreement (κ of 0·32; 88% full agreement).[7] In our study, only diagnoses received during unplanned (i.e. emergency) visits were included in the analyses. This included all visits – both at hospitals, emergency rooms, and specialist outpatient care – that were not made by prior appointment. Diagnoses received during planned visits (i.e. follow-ups and referrals) were excluded. Although this is a more conservative approach, this measure was used to avoid outcome overestimation, as the diagnosis that is the reason for treatment initiation may also be coded during follow-ups and referrals regardless of current symptoms. In sensitivity analyses, we included all visits (i.e. both emergency and planned visits) due to depressive disorders. Missing data in The Swedish Patient Register is around 1% for inpatient treatment, and around 3% for outpatient treatment.[3] In addition, information on deaths from suicide was collected from the Cause of Death Register, a register of all deaths in Sweden, where the underlying cause is specified in 96% of the cases.[1]

Crimes

We tested for associations with examined two other outcomes related to impulsivity; arrests for violent and non-violent crimes, extracted from the Register of Persons Suspected of Offences.[8] We used arrests, rather than convictions, as investigations may dropped by the prosecution, such as when an individual has committed several crimes.[9]

Primary care diagnoses

In sensitivity analyses of depressive disorders, we included only those individuals in the main cohort who had been prescribed statins within Stockholm County. We used data from the Stockholm Primary Care Register from 2006 to 2013 to examine depressive disorders diagnosed in primary care in Stockholm County. This register includes all diagnoses in primary care in the Stockholm County (with a population of over 2 million individuals, i.e. one-fifth of Sweden's population).[4]

Other medications

In sensitivity analyses, we adjusted for treatment with antidepressant medications (ATC: N06A), collected from the Swedish Prescribed Drug Register.[5] Antidepressant treatment periods were defined in the same manner as statin treatment periods (i.e. at least two consecutive dispenses within six months). Antidepressant medications were further stratified into two categories; SSRIs (ATC: N06AB), and all other antidepressants (ATC: N06AA, N06AC, N06AF, N06AG, N06AX). In further sensitivity analyses, we excluded individuals who had also been treated with other medications used for treating cardiovascular diseases who have also been linked to depression;[10,11] β blockers (ATC C07) and angiotensin-converting enzyme (ACE) inhibitors (ATC C09A) (collected from the Swedish Prescribed Drug Register).[5]

Defined Daily Doses

We calculated the defined daily dose exposure in the following way: We collected information on the number of packages for each filled prescription, and on the total amount of defined daily dose specified for each of package from the Swedish Prescribed Drug Register.[5] We multiplied these two variables (i.e. number of packages x total defined daily dose for each package) to calculate the total defined daily dose for each filled prescription. We

then divided this sum by the number of days until the next filled prescription to calculate the mean defined daily dose exposure during this period. We divided each defined daily dose exposure into one of three mutually exclusive categories; low dose (<1 defined daily dose), moderate dose (1-2 defined daily doses), and high dose (>2 defined daily doses), and we compared each treatment interval category to intervals with no use. Using this measure, we identified 26,638,345 treatment intervals. Of those, 60.2% (16,049,126) of the treatment intervals were defined as low exposure, 31.3% (8,338,040) as medium exposure, and 8.5% (2,251,179) as high exposure.

Negative controls
We used two negative controls to test for non-specific treatment effects; thiazide diuretics for systemic use (ATC: C03A) and antihistamines for systemic use (ATC: R06A). The choice of these medications was determined on the basis of theoretical reasons; because they can also be taken daily (like statins), and they are not known to be associated with depression or other psychiatric conditions. Large-scale data do not suggest an association between thiazide diuretics and anxiety or depression.[12,13] Data examining the association between antihistamines for systemic use and neuropsychiatric outcomes is scarce and methodologically limited, precluding a causal association.[14] Information was collected from the Swedish Prescribed Drug Register, [5] and both medications were used as independent exposures in the statins cohort (i.e. regardless of concurrent statin use), and treatment periods were defined in the same manner as statin treatment periods (i.e. at least two dispenses within six months).

Stratified Cox proportional hazards regression
We used stratified Cox proportional hazards regression to examine associations between medications and outcomes.[15] In this model, each individual is entered as a separate stratum in the analysis and serves as his/her own control. The obtained hazard ratio is thus adjusted for (i.e. stratified by) all time-invariant confounders within each individual. In the analyses, all observable time is divided into treatment and non-treatment periods, and each time-to-event is treated as a distinct observation. If outcomes are experienced during a period, this period is further split into the period before the first outcome, period(s) between outcomes, and period after the last outcome. Time-at-risk is measured from the start of all periods, thus accounting for recurrent events. Because the covariates in the stratified Cox proportional hazards regression are time-varying, we did not test for the proportional hazards assumption.[16-18] Because the effect of medication may vary with sex and/or age, we tested for this interaction by introducing two interaction terms as covariates in the model (sex*med and age*med), to examine if further analyses stratified by sex or age were necessary. Results showed no statistically differences (sex: p=.2335; age: p=.2459), and we did therefore not stratify analyses by sex or age.

Appendix Figure 1. Sensitivity analyses of within-individual associations between periods of statins and neuropsychiatric outcomes, by statin class

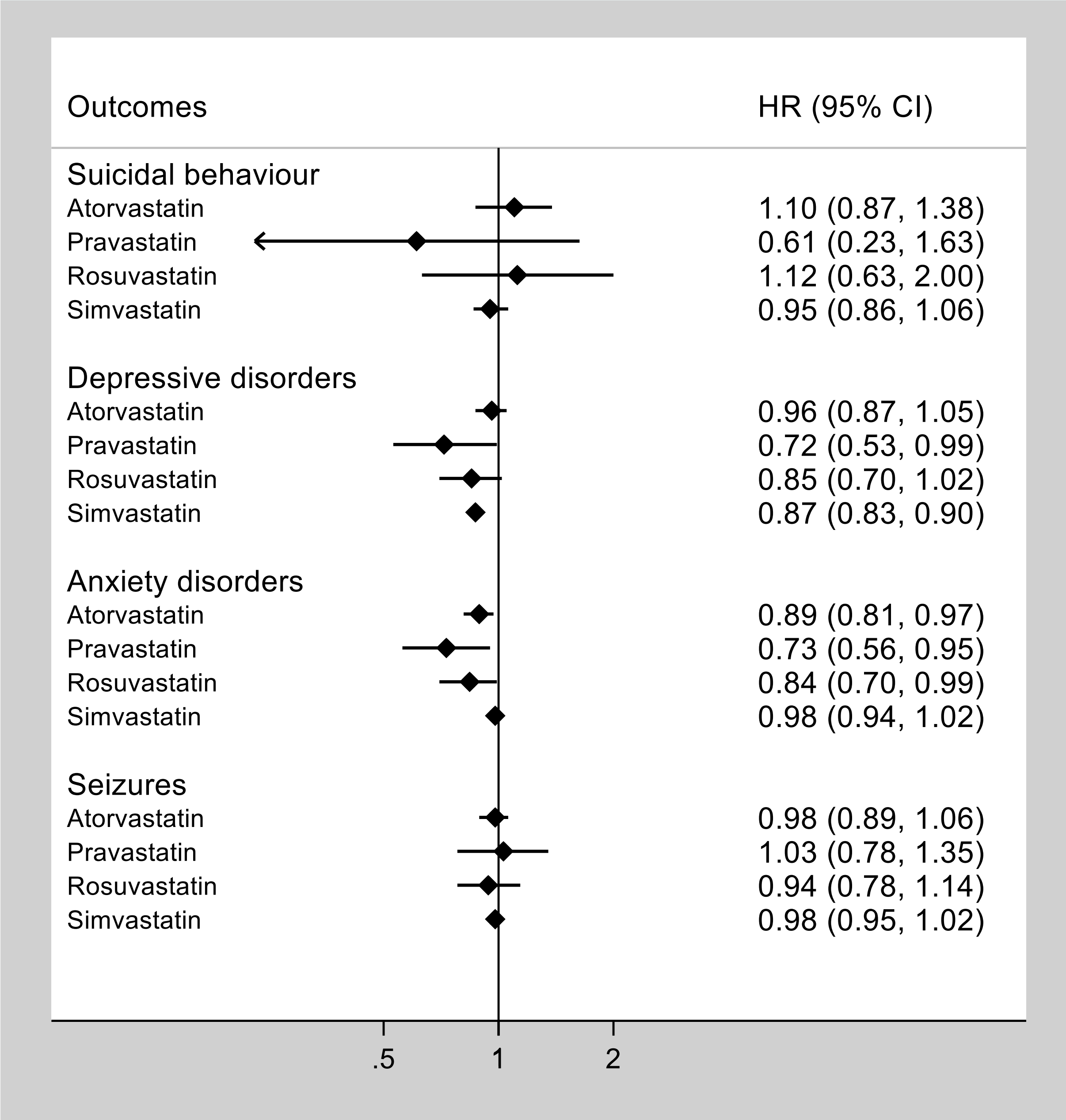

Note: Number in each sample and number of events for each statin class are presented in Appendix Table 1.

Appendix Table 1. Descriptive data for individuals dispensed statins during the study period (2006-13) by statin class

	**Atorvastatin (n=244 482)**	**Pravastatin (n=41 012)**	**Rosuvastatin (n=54 080)**	**Simvastatin (n=1 006 490)**
**Sex**				
Males	136 500 (55·8%)	20 268 (49·4%)	24 948 (46·1%)	546 761 (54·3%)
Females	107 982 (44·2%)	20 744 (50·6%)	29 132 (53·9%)	459 729 (45·7%)
**Age at the start of the study period**				
<40	6 830 (2·8%)	590 (1·4%)	1 902 (3·5%)	26 493 (2·6%)
40-49	23 331 (9·5%)	2 022 (4·9%)	6 026 (11·1%)	86 693 (8·6%)
50-59	61 740 (25·3%)	7 231 (17·6%)	15 643 (28·9%)	219 777 (21·8%)
60-69	88 390 (36·2%)	14 363 (35·0%)	20 272 (37·5%)	327 281 (32·5%)
70-79	51 216 (21·0%)	12 032 (29·3%)	8 937 (16·5%)	247 198 (24·6%)
≥80	12 975 (5·3%)	4 774 (11·6%)	1 300 (2·4%)	99 048 (9·8%)
**Outcomes during the study period**				
Self-injurious or suicidal behaviour*	1 295 (0·5%)	0·4% (167)	262 (0·5%)	5 573 (0·6%)
Depressive disorders	5 955 (2·4%)	943 (2·3%)	1 203 (2·2%)	26 644 (2·7%)
Anxiety disorders	6 201 (2·5%)	905 (2·2%)	1 400 (2·6%)	25 219 (2·5%)
Seizures	4 364 (1·8%)	690 (1·7%)	768 (1·4%)	21 269 (2·1%)
**Number of events during the study period**				
Self-injurious or suicidal behaviour*	1 604	242	107	6 209
Depressive disorders	7 806	1 597	813	35 172
Anxiety disorders	8 820	1 870	894	37 287
Seizures	7 337	1 220	790	39 046

* Includes suicide attempt and death from suicide

Appendix Table 2. Sensitivity analyses of within-individual associations between periods of statins and crime outcomes

Crime outcomes	Number in sample	Number of events	Hazard ratio (95% CI)
Arrests for violent crime	1 149 384	8 543	0·81 (0·73-0·89)
Arrests for non-violent crime	1 149 384	77 167	0·90 (0·88-0·93)

REFERENCES

1. Brooke HL, Talback M, Hornblad J, et al. The Swedish cause of death register. *Eur J Epidemiol* 2017; **32**: 765-73.
2. Ludvigsson JF, Almqvist C, Bonamy AK, et al. Registers of the Swedish total population and their use in medical research. *Eur J Epidemiol* 2016; **31**: 125-36.
3. Ludvigsson JF, Andersson E, Ekbom A, et al. External review and validation of the Swedish national inpatient register. *BMC Public Health* 2011; **11**: 450.
4. Wandell P, Carlsson AC, Wettermark B, Lord G, Cars T, Ljunggren G. Most common diseases diagnosed in primary care in Stockholm, Sweden, in 2011. *Fam Pract* 2013; **30**: 506-13.
5. Wettermark B, Hammar N, Fored CM, et al. The new Swedish Prescribed Drug Register--opportunities for pharmacoepidemiological research and experience from the first six months. *Pharmacoepidemiol Drug Saf* 2007; **16**: 726-35.
6. Ludvigsson JF, Otterblad-Olausson P, Pettersson BU, Ekbom A. The Swedish personal identity number: possibilities and pitfalls in healthcare and medical research. *Eur J Epidemiol* 2009; **24**: 659-67.
7. Fazel S, Wolf A, Chang Z, Larsson H, Goodwin GM, Lichtenstein P. Depression and violence: a Swedish population study. *Lancet Psychiat* 2015; **2**: 224-32.
8. National Council for Crime Prevention [Brottsförebyggande Rådet]. Criminal statistics, Official Statistics of Sweden 2014 [Kriminalstatistik 2014] Stockholm: BRÅ Rapport 2015:16 ISSN 1100-667; 2015.
9. The Swedish Government Official Reports 2006:30 [Statens Offentliga Utredningar 2006:30]. Is justice fair? Ten perspectives on discrimination of ethnic and religious minorities in the judicial system [Är rättvisan rättvis? Tio perspektiv på diskriminering av etniska och religiösa minoriteter inom rättssystemet] Stockholm: SOU 2006:30 ISSN 0375-250X; 2006.
10. Battes LC, Pedersen SS, Oemrawsingh RM, et al. Beta blocker therapy is associated with reduced depressive symptoms 12 months post percutaneous coronary intervention. *J Affect Disord* 2012; **136**: 751-7.
11. Vian J, Pereira C, Chavarria V, et al. The renin-angiotensin system: a possible new target for depression. *BMC Med* 2017; **15**: 144.
12. Suchard MA, Schuemie MJ, Krumholz HM, et al. Comprehensive comparative effectiveness and safety of first-line antihypertensive drug classes: a systematic, multinational, large-scale analysis. *Lancet* 2019; **394**: 1816-26.
13. Boal AH, Smith DJ, McCallum L, et al. Monotherapy with Major antihypertensive drug classes and risk of hospital admissions for mood disorders. *Hypertension* 2016; **68**: 1132-8.
14. Boer J, Ederveen E, Grundmark B. Desloratadine and depression, a drug safety signal based on worldwide spontaneous reporting of side effects. *Ups J Med Sci* 2018; **123**: 174-8.
15. Lichtenstein P, Halldner L, Zetterqvist J, et al. Medication for attention deficit-hyperactivity disorder and criminality. *N Engl J Med* 2012; **367**: 2006-14.
16. Allison PD. Fixed-Effects Partial Likelihood for Repeated Events. *Sociol Methods Res* 1996; **25**: 207-22.
17. Allison PD. Fixed effects regression models: SAGE publications; 2009.
18. Whitaker HJ, Hocine MN, Farrington CP. The methodology of self-controlled case series studies. *Stat Methods Med Res* 2009; **18**: 7-26.

STROBE

STROBE Statement—Checklist of items that should be included in reports of ***cohort studies***

	Item No	Recommendation	Page No
**Title and abstract**	1	(*a*) Indicate the study's design with a commonly used term in the title or the abstract<br>(*b*) Provide in the abstract an informative and balanced summary of what was done and what was found	(a) Title + Abstract p. 1<br>(b) p. 1
**Introduction**			
Background/rationale	2	Explain the scientific background and rationale for the investigation being reported	pp. 1-2
Objectives	3	State specific objectives, including any prespecified hypotheses	p. 2
**Methods**			
Study design	4	Present key elements of study design early in the paper	p. 2
Setting	5	Describe the setting, locations, and relevant dates, including periods of recruitment, exposure, follow-up, and data collection	p. 2-3 + Appendix pp. 2-3
Participants	6	(*a*) Give the eligibility criteria, and the sources and methods of selection of participants. Describe methods of follow-up<br>(*b*) For matched studies, give matching criteria and number of exposed and unexposed	(a) pp. 2-3 + Appendix pp. 2-3<br>(b) n/a (self-controlled case series)
Variables	7	Clearly define all outcomes, exposures, predictors, potential confounders, and effect modifiers. Give diagnostic criteria, if applicable	pp. 2-3 + Appendix pp. 2-3
Data sources/ measurement	8*	For each variable of interest, give sources of data and details of methods of assessment (measurement). Describe comparability of assessment methods if there is more than one group	pp. 2-3 + Appendix pp. 2-3
Bias	9	Describe any efforts to address potential sources of bias	pp. 2-4 + Appendix pp. 2-3
Study size	10	Explain how the study size was arrived at	p. 3
Quantitative variables	11	Explain how quantitative variables were handled in the analyses. If applicable, describe which groupings were chosen and why	pp. 3-4 + Appendix p. 3
Statistical methods	12	(*a*) Describe all statistical methods, including those used to control for confounding<br>(*b*) Describe any methods used to examine subgroups and interactions<br>(*c*) Explain how missing data were addressed<br>(*d*) If applicable, explain how loss to follow-up was addressed<br>(*e*) Describe any sensitivity analyses	(a) pp. 3-4 + Appendix p. 3<br>(b) pp. 3-4 + Appendix pp. 2-3<br>(c) pp. 3-4 + Appendix pp. 2-3<br>(d) p. 3<br>(e) pp. 3-4 + Appendix pp.2-3
**Results**			
Participants	13*	(a) Report numbers of individuals at each stage of study—eg numbers potentially eligible, examined for eligibility, confirmed eligible, included in the study, completing follow-up, and analysed<br>(b) Give reasons for non-participation at each stage<br>(c) Consider use of a flow diagram	(a) pp. 5-6 + Tables 1-2, Appendix Tables 1-2<br>(b) n/a<br>(c) n/a
Descriptive data	14*	(a) Give characteristics of study participants (eg demographic, clinical, social) and information on exposures and potential confounders<br>(b) Indicate number of participants with missing data for each variable of interest<br>(c) Summarise follow-up time (eg, average and total amount)	(a) p. 5 + Table 1<br>(b) Appendix p. 2<br>(c) p. 9-10
Outcome data	15*	Report numbers of outcome events or summary measures over time	pp. 5-6 + Tables 1-2+ Appendix Tables 1-2
Main results	16	(*a*) Give unadjusted estimates and, if applicable, confounder-adjusted estimates and their precision (eg, 95% confidence interval). Make clear which confounders were adjusted for and why they were included<br>(*b*) Report category boundaries when continuous variables were categorized<br>(*c*) If relevant, consider translating estimates of relative risk into absolute risk for a meaningful time period	(a) pp. 5 + Figure 1<br>(b) n/a<br>(c) n/a
Other analyses	17	Report other analyses done—eg analyses of subgroups and interactions, and sensitivity analyses	(a) pp. 5-6 + Table 2 + Appendix Figure 1 + Appendix Table 2
**Discussion**			
Key results	18	Summarise key results with reference to study objectives	p. 6
Limitations	19	Discuss limitations of the study, taking into account sources of potential bias or imprecision. Discuss both direction and magnitude of any potential bias	pp. 6-8
Interpretation	20	Give a cautious overall interpretation of results considering objectives, limitations, multiplicity of analyses, results from similar studies, and other relevant evidence	pp. 6-8
Generalisability	21	Discuss the generalisability (external validity) of the study results	p. 8
**Other information**			

Funding	22	Give the source of funding and the role of the funders for the present study and, if applicable, for the original study on which the present article is based	pp. 4-5, 8

*Give information separately for exposed and unexposed groups.

Note: An Explanation and Elaboration article discusses each checklist item and gives methodological background and published examples of transparent reporting. The STROBE checklist is best used in conjunction with this article (freely available on the Web sites of PLoS Medicine at http://www.plosmedicine.org/, Annals of Internal Medicine at http://www.annals.org/, and Epidemiology at http://www.epidem.com/). Information on the STROBE Initiative is available at http://www.strobe-statement.org.
